# surviveR: a flexible shiny application for patient survival analysis

**DOI:** 10.1038/s41598-023-48894-9

**Published:** 2023-12-13

**Authors:** Tamas Sessler, Gerard P. Quinn, Mark Wappett, Emily Rogan, David Sharkey, Baharak Ahmaderaghi, Mark Lawler, Daniel B. Longley, Simon S. McDade

**Affiliations:** Patrick G. Johnston Centre for Cancer Research, Queen’s University Belfast, Belfast, UK

**Keywords:** Computational biology and bioinformatics, Computational platforms and environments, Data integration, Data mining, Software

## Abstract

Kaplan–Meier (KM) survival analyses based on complex patient categorization due to the burgeoning volumes of genomic, molecular and phenotypic data, are an increasingly important aspect of the biomedical researcher’s toolkit. Commercial statistics and graphing packages for such analyses are functionally limited, whereas open-source tools have a high barrier-to-entry in terms of understanding of methodologies and computational expertise. We developed surviveR to address this unmet need for a survival analysis tool that can enable users with limited computational expertise to conduct routine but complex analyses. surviveR is a cloud-based Shiny application, that addresses our identified unmet need for an easy-to-use web-based tool that can plot and analyse survival based datasets. Integrated customization options allows a user with limited computational expertise to easily filter patients to enable custom cohort generation, automatically calculate log-rank test and Cox hazard ratios. Continuous datasets can be integrated, such as RNA or protein expression measurements which can be then used as categories for survival plotting. We further demonstrate the utility through exemplifying its application to a clinically relevant colorectal cancer patient dataset. surviveR is a cloud-based web application available at https://generatr.qub.ac.uk/app/surviveR, that can be used by non-experts users to perform complex custom survival analysis.

## Introduction

The predominant method for analysing survival in a patient cohort is calculating the changes in the proportion of living individuals over time, which can be visualised using Kaplan-Meyer (KM) plots^1^. An important consideration in KM analysis is that such patient data almost always contains incomplete observations; therefore, survival analysis must be able to deal with data for differing survival times when not all the individuals complete the study. The complexities of plotting such probability-based graphs with incomplete observations result in a reliance on commercial statistics or graphing software that are not designed for complex stepwise patient filtering and groupings. The difficult analyses are becoming a day-to-day task for life sciences and clinical researchers who aim to exploit the burgeoning amounts of data being generated from samples derived from patients with cancer and other diseases.

While these types of analyses are possible with open-source R-based computational packages, utilisation of such tools for complex analyses requires significant computational expertise. Moreover, due to exponentially increasing accumulation of genomic and proteomic data, coupled with other quantitative and clinicopathological data associated with patient samples, it is increasingly important to understand the distribution of continuous markers and to be able to discretise them. Continuous values such as expression data cannot easily be integrated into existing survival platforms. surviveR allows the user to easily integrate these with other categorical variables (e.g., mutation, staging and transcriptomic classifiers) to enable their inclusion in survival analyses. Additionally, there are currently limited user-friendly, freely available software and useful tools such as KMPlot^2^ are more focused on analysis of large public datasets such as TCGA, with limited functionalities needed by researchers who lack sufficient computational skills to better analyse clinical datasets. To address this unmet need, we developed surviveR, a flexible cloud-based Shiny app for user-friendly survival analysis.

## Methods

**Figure 1:**
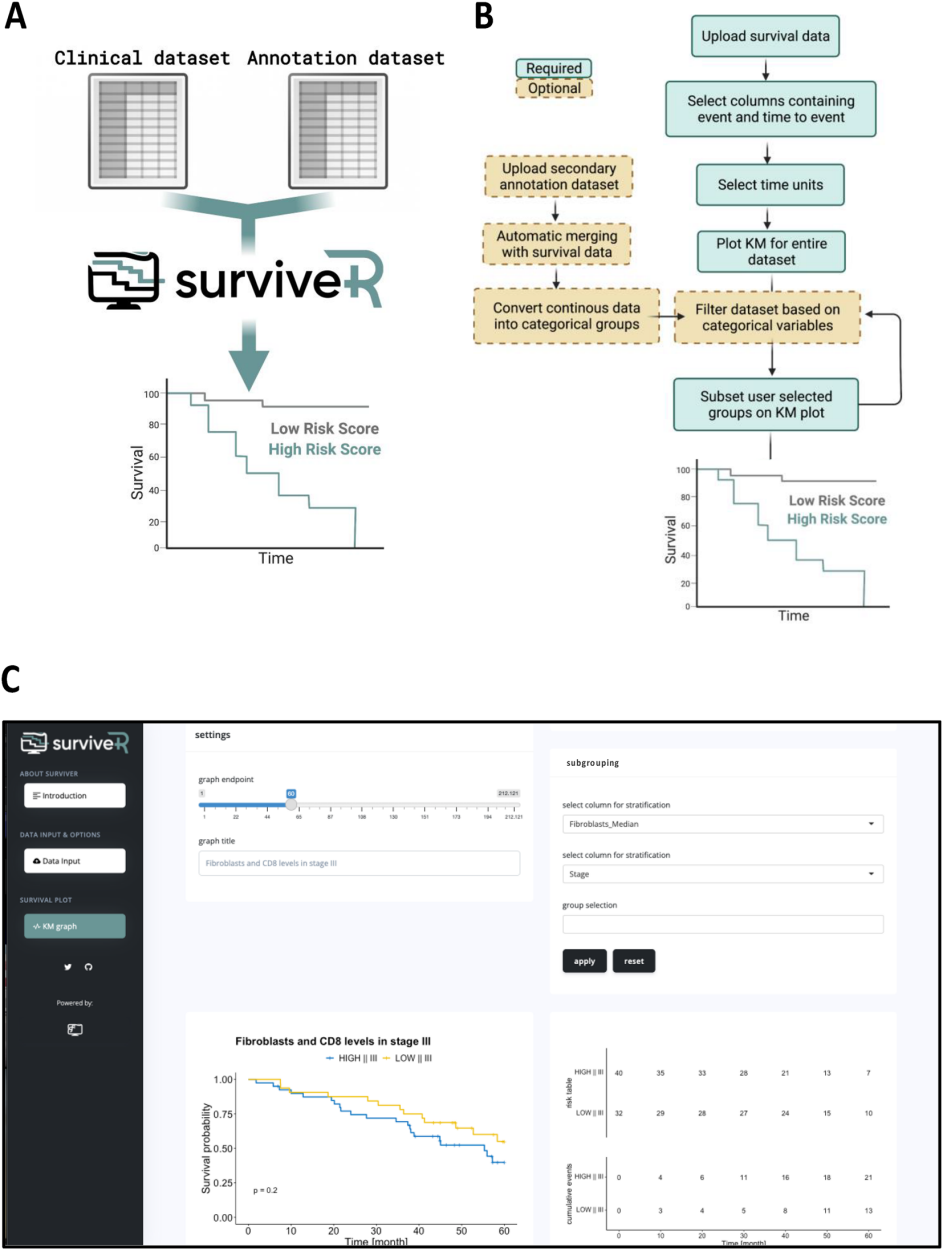
Processes and functionality in the backend of the survive shiny application. (**A**) Visual abstract of surviveR application (**B**) Screenshot of the GUI of surviveR KM graph page. (**C**) Schematic overview of survive architecture and sub-functions.

surviveR is developed in the R-environment^3^ (R Core Team, 2014) using Shiny to allow the R code to run within a HTML and JavaScript framework^4^. It utilises Shiny packages (shiny, argonDash, shinyjs)^4–6^ to translate R code into an interactive web application. surviveR is designed with a user-friendly graphical user interface with detailed description of applied methods and instructions for use by non-expert users (Fig. 1 and Fig. S1). The data reading and handling core is based on the processes data.table, dplyr and DT^7^ packages while the survival data is analysed and graphed through the functionalities of the survminer^8^, survival^9^ and ggplot2 R packages^10^. Cox hazard ratio (HR) is expressed as (exp(coef)), while the inverse HR (exp(-coef)), the reference interval (upper.95 and lower.95) and the p value are also reported. surviveR provides additional options such as continuous data integration (e.g., conversion of the expression of a specific gene split by median to provide High and Low expression categories), filtering and multiple levels of grouping. These new groupings can then be plotted directly within surviveR.

All figures and tables are downloadable in .pdf or .csv format through a download button under each table or plot. surviveR is freely available at: generatr.qub.ac.uk/app/surviveR.

In the worked example, the transcriptionally derived annotation of gene expression microarray data from our previously described CRUK taxonomy cohort^11^ was further annotated with our published classifieR^c^ app^12^ that is freely available https://generatr.qub.ac.uk/app/classifieRc for consensus molecular subtypes (CMS)^13^, Microenvironment Cell Populations-counter (MCP-counter)^14^ for Fibroblasts. Microarray profiling and patient data is available from GEO with accession GSE103479**.**

## Results

The surviveR app has 3 tabs (Fig. 1C):

1. The Introduction tab describes the information underlying the various models used for KM analyses within surviveR.
2. The Data input tab walks users through file upload, end-point definition needed for survival and risk analysis and optional settings such as time unit changes and data dichotomization.
3. The results are displayed on the KM survival curve tab containing graphical and statistical outputs (Fig. 1C and S1).

surviveR can read-in a range of standard (delimited) data tables (e.g., .csv) where each row must represent a patient and each column a patient descriptor (e.g., sex, treatment…), categorical or continuous molecular characteristic (e.g., mutation status, IHC-score, gene expression level). Once uploaded, the user is required to select/define the columns containing (i) event of interest (e.g., progression, death) and time to event. This is necessary to allow flexibility between datasets since such data is often differently annotated from user to user and further allows flexible selection of different endpoints in addition to live/dead survival. The default setting in surviveR assumes that the event time is given in months, however the input and the graphed time format can be changed/re-calculated using a drop-down menu. Once all options are selected, users move onto the KM survival curve tab by pressing the “Graph Me” button, and surviveR automatically displays a KM plot comparing defined groups with an estimated log-rank test p-value using a 5-year endpoint (default, can be changed using a slider), along with a risk and a cumulative event table as illustrated in Fig. 1C.

SurviveR has built-in capability to carry out multiple data pre-filtering since users often want to carry out survival analysis from a defined sub-cohort (e.g., Stage-II patient only). This is achieved iteratively by selecting the appropriate column and value/s from a drop-down menu, where for example, a user could select only patients of a particular stage and/or with a mutation in a specific gene. Once any pre-filtration is completed, grouping of remaining patients is conducted by selecting up to two categorical variable columns, which by default plots a KM-curve showing all sub-groups (or combinations of variables) separately. If the selected variable for subgrouping results in more than two groups, further flexibility is provided by the option to visualise specific subsets versus each other or the “rest” of the cohort, where patient data belonging to the non-selected categories are merged and visualised as one group (Fig. S1D). The significance of differences between two or multiple patient subgroups is estimated automatically using a pairwise log-rank test and Cox proportional hazard regression model. The pairwise log-rank test automatically calculates a multiple comparison, returning a p-value between all shown subgroups, while the Cox proportional hazard regression model is calculated using the selected group as a reference cohort from a drop-down menu (Fig. 1C).

**Figure 2:**
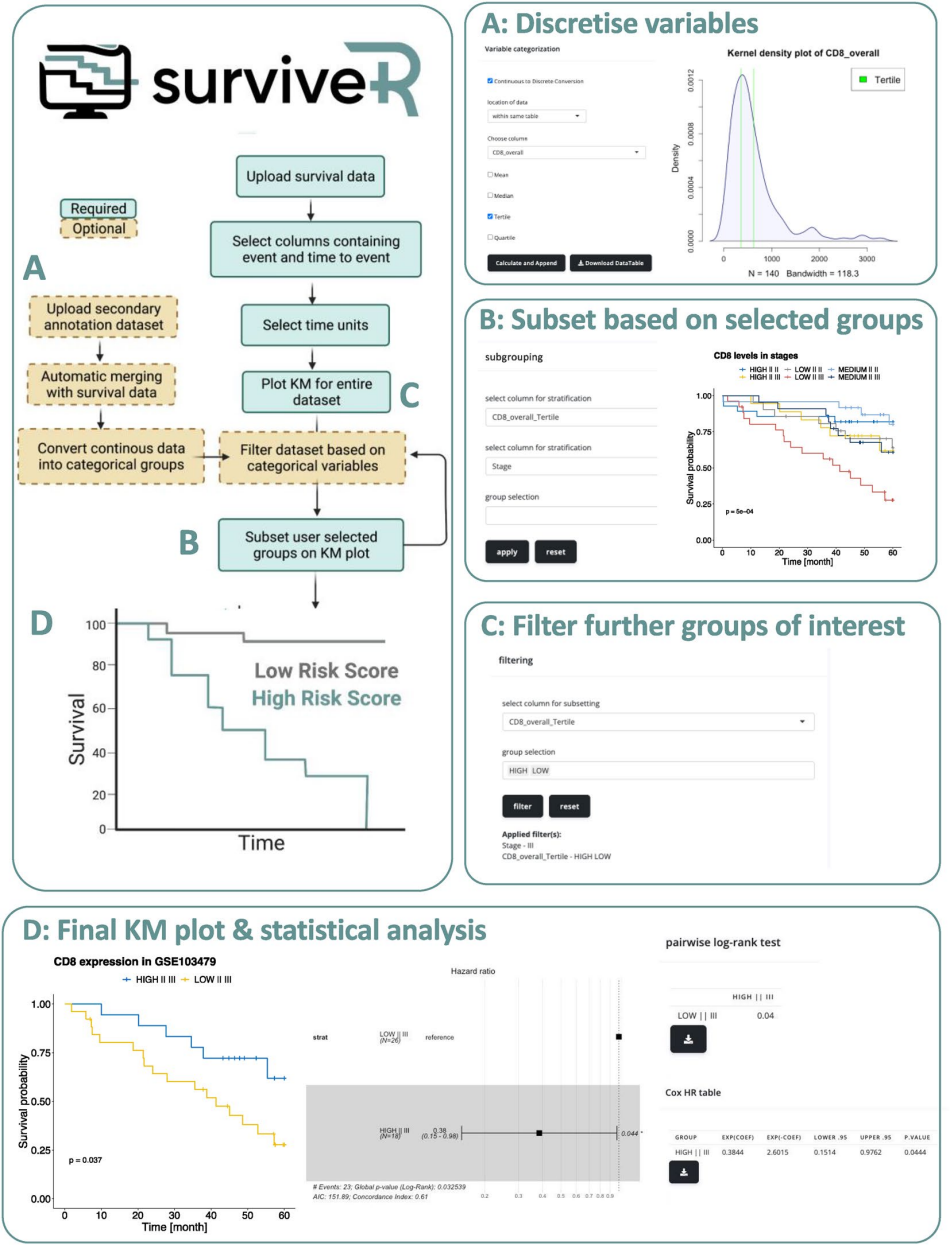
Example usage of the surviveR application with continuous variable stratification and generation of a KM plot using the GSE103479 dataset. (**A**) Conversion of a continuous variable (CD8_overall) into a categorial variable using a tertile split. This will generate 3 categories Low, Medium and High for that column. (**B**) Using the generated categories from **(A**), the KM plot can be split based on the CD8_Overall_Tertile column. (**C**) Data plotted on the KM plot can then be filtered to only show a user groups of interest (High and Low CD8_Overall_Tertile). This can be applied before or after generation of the KM plot. (**D**) Final KM plot showing survival across 5 years on the GSE103479 dataset, hazard ratio and log-rank and Cox HR statistical analysis.

KM analysis, by definition requires patients to be subgrouped using categorical values; thus, in order to enable use of continuous variables, the surviveR data input tab is additionally enabled with the capability to easily convert continuous data (e.g., gene expression values, protein expression, immune cell counts, etc.) into discrete categorical variables in the data input tab through various conversions: (i) High-Low based on mean or median, (ii) tertile, (iii) quartile or (iv) binary division by selecting a custom value. For each continuous variable to be categorised, surviveR automatically plots a Kernel frequency distribution plot along with the cut-off values of the chosen methods of transformation to help guide selection of the most appropriate methods of categorisation (Fig. 2A–D). This is achieved either for a variable encoded within the uploaded survival data table or it can be extracted from an additional delimited table (e.g., RNA-seq data matrix, MCP-Counter inferred measure of cellular composition). In the latter case, the uploaded additional data should have a column containing the patient IDs matching the original uploaded table enclosing the survival information.

### Use-case Reanalysis of GSE103479 CRC data from Allen et al. ^11^ (Fig. S1)

As an example of a complex survival analyses of a stage 2/3 colorectal cancer (CRC) cohort the analysis of which we recently reported ^11^ (GSE103479) can be reproduced in minutes using surviveR as shown in Supplementary Fig. S1. Here we show in detail how to use surviveR to demonstrate that the levels of CD8+ tumour infiltrating lymphocytes (TILs) (quantified by IHC) in Stage III CRC, transcriptionally-inferred CRC specific consensus molecular subtypes (CMS)^13^ group patients (inferred using our recently published classifieR^C^ app^12^ can be prognostic/predictive of patient outcomes with respect to untreated and treated CRC patients.

After uploading the clinical data in surviveR, trichotomisation (for example) of the CD8 levels can be achieved under “Variable categorization” by ticking the “Continuous to Discreet Conversion” checkbox. If the data to be transformed is already contained within the same uploaded table as the clinical data, where the user only needs to select the appropriate column name and the type of cut-off value they wish to apply (e.g., Median, Mean, Tertile and Quartile). Once a column is selected, a graph automatically appears (where) showing the Kernel distribution of the chosen data with the selected cut-off values (Fig. S1A). The newly calculated values can be attached to our table by clicking the “Calculate and Append” button. The column that contains patient survival time (OverallSurvival) and patient outcome (Alive_dead) can be selected from a dropdown menu (where the surviving patients are denoted as “Alive” and deceased patients are marked as “Dead”); the KM plot can be visualised by clicking the “Graph Me” button (Fig. S1A,B). The dynamic plot is then rendered on the “KM graph” tab of the application.

To investigate how stages and CD8 levels affect patient survival the user can select these columns in the dropdown menu under “Subgrouping” on the KM graph tab. Since this dataset contains both Stage II and Stage III patients, to filter out Stage II patients, we can select Stage III patients under the group selection option or using the “filtering” options where we first need to select the column to use as filter and the value(s) we would like to keep. Using consecutive filtering criteria, we can refine the visualised patient cohort to contain only Stage III patients and subgroup these patients using the CMS subgroup annotation. Filtering out Stage II patients and subgrouping them by the four CMS subtypes (1–4), we can demonstrate that as in Allen et al.^11^ that CD8 levels have a significant impact on outcome for patients with Stage III CMS2 tumours in this cohort.

### Use-case #2 Survival data integration with other multi-omic datasets (Fig. S2)

surviveR is designed to easily receive the input from other data analysis tools. We and others have previously demonstrated that stromal content and cancer-associated fibroblasts (CAF) are associated with poor outcomes in colorectal cancers and it is possible to estimate the levels of Fibroblast stroma using MCP-counter^14^ that is easily accessible to non-computational user in our recently released classifieR^c^ app^12^. The GSE103479 dataset was annotated with MCP-Counter cellular composition scores in classifieR^c^ and uploaded to surviveR. These values were then used to dichotomise patients as high or low for inferred CD8 expression from mRNA or Fibroblast stroma levels by selecting the Median cut-off method under the “Continuous to Discreet Conversion” section (Fig. S2A). After selecting the required patient time and outcome settings as previously described, we subgrouped the patients’ KM plots by the newly calculated Fibroblast_median or CD8_median columns (Fig. S2A) and further filtered the data with Stage of the disease (Fig. S2B,C). While as previously observed low levels of CD8 correlates with worse prognosis, patients with high levels of fibroblasts have significantly worse outcomes, observable when filtering the cohort by Stage III patient (Fig. S2C). Interestingly, when subgrouping for Fibroblast and CD8 levels in the different stages, high levels of Fibroblast in Stage II patients correlated with worse overall survival only in patients with low levels of CD8 in the tumour (Fig. S2D), while in Stage III patients high CD8 expression causes better prognosis only when associated with low levels of Fibroblasts and high CD8 expression (Fig. S2D).

## Conclusion

The increasing volume and variety of clinical, molecular and phenotypic data linked to patient samples presents significant challenges for increasingly complex analysis of correlation with patient outcomes. While there are available online and offline tools that would render the analysis of custom clinical datasets possible, they are difficult to use and/or unable to perform complex tasks such as subgrouping and seamless integration of continuous variables. Here, we report surviveR, a freely available, easy to use Shiny app that empowers end users with limited bioinformatics expertise to analyse survival data and to identify novel prognostic biomarkers.

A major advantage of the surviveR application over other tools is the ability to integrate continuous variables present in either the same table or in an external dataset. Allowing the application the use of different cut-off values and enables the user to easily combine clinical data with different quantitative multi-omic datasets (e.g., gene expression). The Manual selection of survival time point, event label and time unit renders surviveR a flexible tool adaptable to different, custom datasets. For complex data analysis, patient sub-cohorts can be selected by multiple consecutive filtering steps and by the selection of up to two descriptors for subgrouping. To help with the data interpretation, surviveR will report risk and cumulative event table, along with calculated p values for log-rank tests and Cox hazard ratio.

In summary we have developed an easy to use, fast and powerful online tool for the analysis of survival data that is able to deal with custom datasets and perform complex analysis, rendering surviveR a highly valuable asset for clinical and biomedical researchers.

### Supplementary Information

Supplementary Figures.

## Data Availability

Microarray profiling and patient data used in paper is available from Gene Expression Omnibus (GEO) with accession GSE103479. surviveR is a cloud-based web application and is available via free login online at https://generatr.qub.ac.uk/app/surviveR.
