# Supplementary material for: surviveR: a flexible shiny application for patient survival analysis
Source: Sci Rep. 2023 Dec 13;13:22093. doi: 10.1038/s41598-023-48894-9 (PMC10716386; doi:10.1038/s41598-023-48894-9)
Supplement: Supplementary file 1 — Supplementary Figures. [file 41598_2023_48894_MOESM1_ESM.pdf]

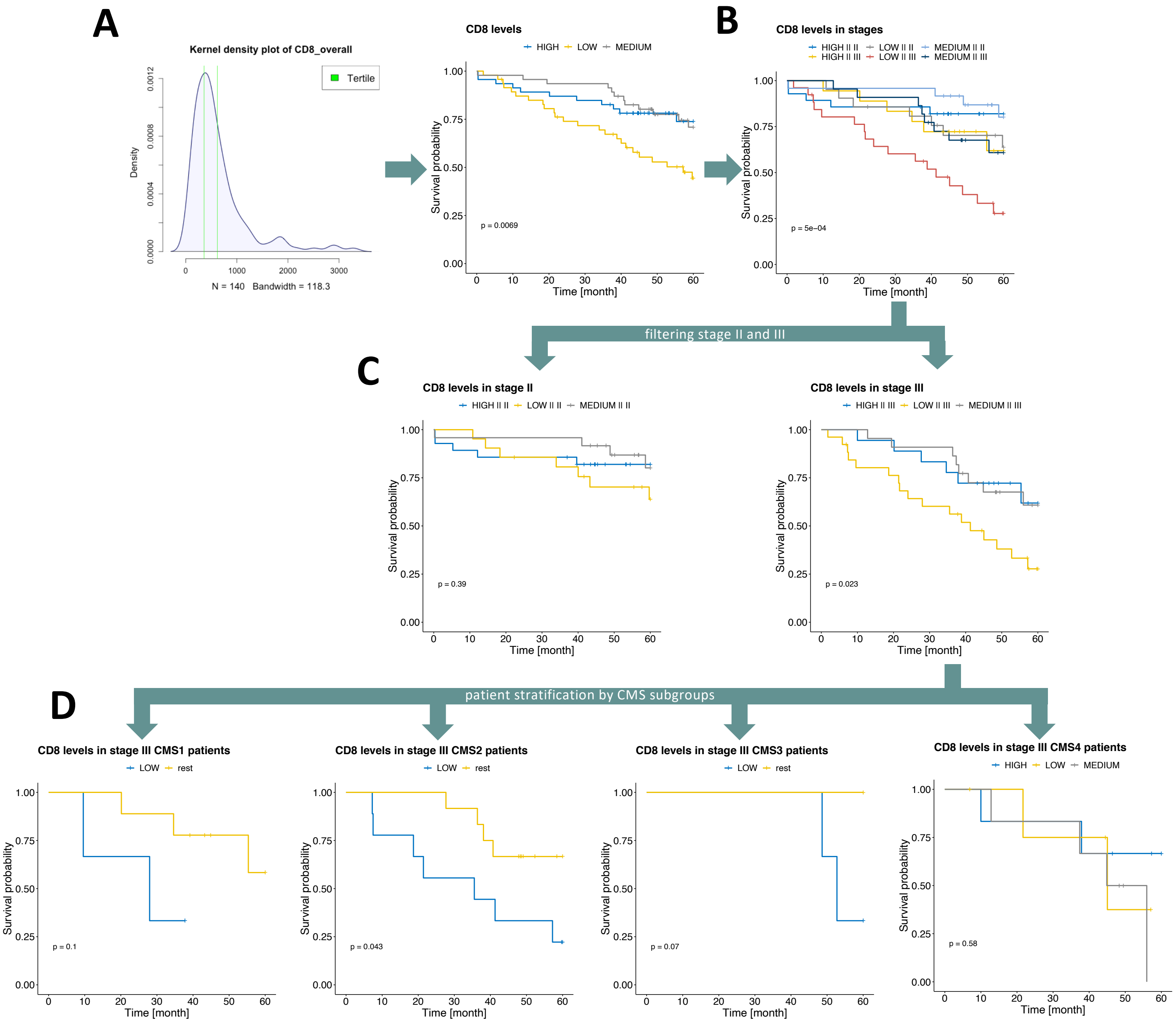

Supplementary Figure 1. Use case 1: Low levels CD8 levels in stage III patients correlates with worse prognosis in CMS2 subgroup demonstrated within the surviveR application. (**A**) As described in Allen et al (JCO Precis Oncol 2018 Jun 13;2018.) the overall CD8 levels in the GSE103479 dataset were used to divide patients into low, medium and high CD8 expressing tumors and the 5-year survival was plotted. (**B**) Patients were further stratified by stage, then visualized separately by filtering stage II (left) or stage III (right) patients. (**C**) As only stage III patients showed significant difference in patient survival by CD8 levels, only this groups was used to separately plot patients with low CD8 expressing tumour staining versus medium and high levels (merged as "rest") (**D**). In conclusion in the GSE103479 cohort, patients with low levels of CD8 staining are significantly more at risk if the patient bears a stage III, CMS2 type colorectal tumor.

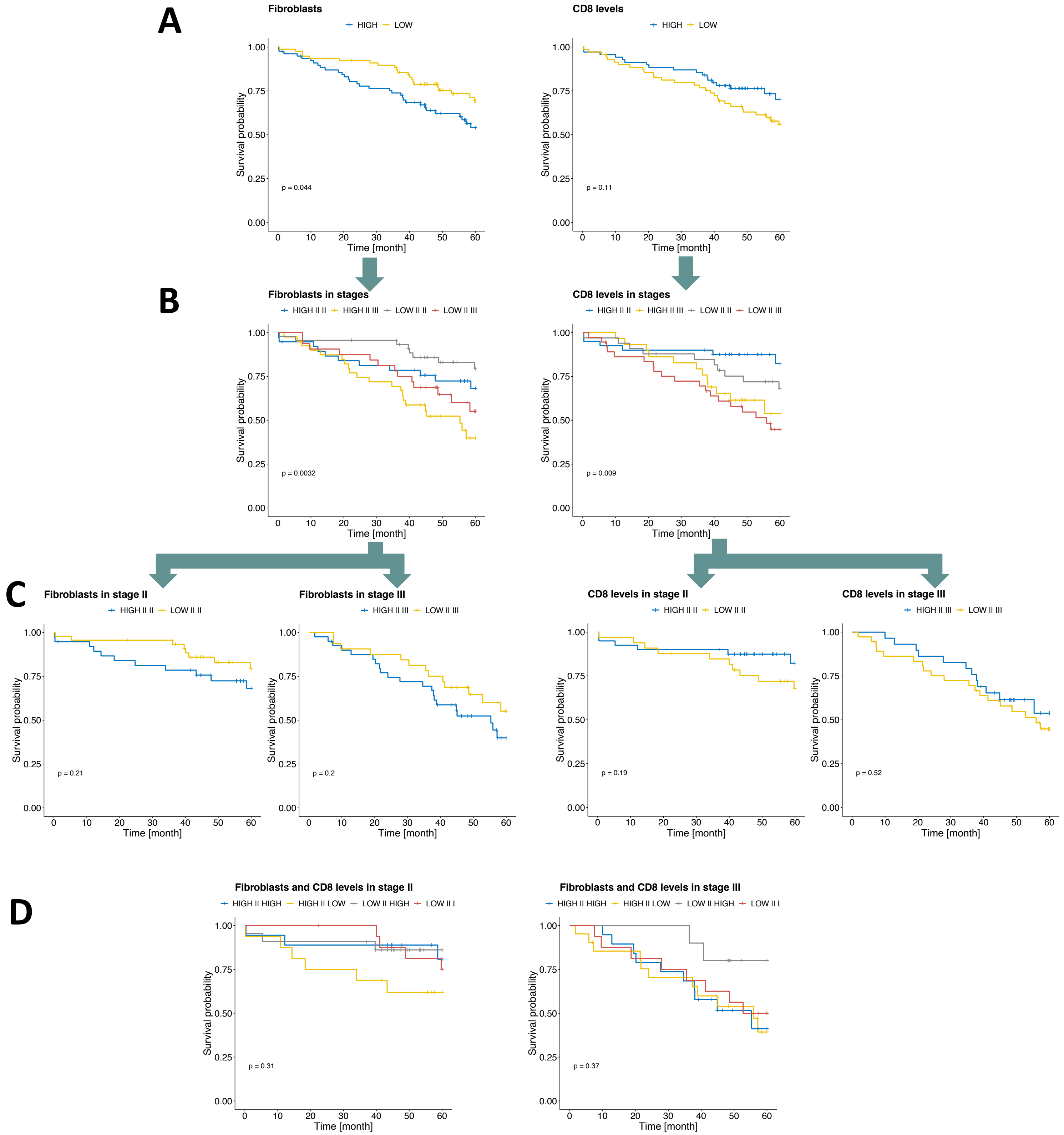

	HIGH || HIGH	HIGH || LOW	LOW || HIGH
HIGH || LOW	0.46	-	-
LOW || HIGH	0.93	0.46	-
LOW || LOW	0.92	0.48	0.92

	HIGH || HIGH	HIGH || LOW	LOW || HIGH
HIGH || LOW	0.98	-	-
LOW || HIGH	0.28	0.28	-
LOW || LOW	0.82	0.82	0.38

Supplementary Figure 2. Use case 2: Demonstrating the ability of surviveR to easily take the input from other data analysis tools such as MCP-counter scores generated by classifieR. (**A**) Creating dichotomised values for CD8 expression and Fibroblast stroma levels by selecting the Median cut-off method under the “Continuous to Discreet Conversion” section. (**B**) After selecting the required patient time and outcome settings as previously, we can stratify the KM plots by selecting the newly calculated Fibroblast_Median or CD8_Median columns alone. (**C**) Data can be then stratified further by TMN staging. (**D**) Combined groups with both fibroblast and CD8 levels combined and plotted in both stage II and stage III disease with the logP value showing statistical difference between the subcohorts of pationets.
